# Differences between persistent and episodic depression in processing novel positive information

**DOI:** 10.1017/S0033291725101530

**Published:** 2025-09-04

**Authors:** Tobias Kube, Edith Rapo, Mimi Houben, Thomas Gärtner, Eva-Lotta Brakemeier, Julia Anna Glombiewski, Winfried Rief

**Affiliations:** 1 Department of Clinical Psychology and Psychotherapy, RPTU Kaiserslautern-Landau, Landau, Germany; 2 Department of Clinical Psychology and Experimental Psychopathology, Goethe University of Frankfurt, Frankfurt, Germany; 3 Department of Clinical Psychology and Psychotherapy, Philipps-University of Marburg, Marburg, Germany; 4 Department of Psychosomatics and Psychotherapy,Schön Klinik Bad Arolsen, Bad Arolsen, Germany; 5 Department of Clinical Psychology and Psychotherapy, University of Greifswald, Greifswald, Germany

**Keywords:** belief updating, depression, early onset, expectation change, information processing, treatment expectation

## Abstract

**Background:**

Research has pointed to important psychopathological differences between persistent and episodic depressive disorders. Here, we tested the hypothesis that people with persistent rather than episodic depression have difficulty revising established expectations in response to novel positive information. In terms of underlying mechanisms, we predicted that these differences between the two subtypes would be related to the engagement in cognitive immunization (i.e. devaluing expectation-disconfirming positive information).

**Methods:**

Prior to their psychotherapeutic treatment, 54 outpatients with persistent depressive disorder and 102 outpatients with episodic major depressive disorder completed an experimental task. In this task, participants watched other patients’ reports of positive effects of psychotherapy. Our primary outcome was change in treatment expectations from before to after watching the positive reports.

**Results:**

Overall, people with persistent depression had lower treatment expectations than people with episodic depression. In addition, they changed their treatment expectations less in response to other patients’ positive reports. This effect was greater for psychotherapy outcome expectations than for role expectations. The lack of expectation change in persistent depression relative to episodic depression was particularly pronounced in a cognitive immunization-promoting experimental condition.

**Conclusions:**

The results indicate that people with persistent depression have difficulty adjusting their treatment expectations in response to positive information on psychotherapy. This may be a risk factor for poor treatment outcome. The results regarding cognitive immunization suggest that for people with persistent depression, slight doubts about the value of information on the positive effects of psychotherapy may be sufficient to prevent them from integrating this information.

## Introduction

In around 20–30% of people who suffer from a major depressive disorder, the symptoms are chronic (Angst, Gamma, Rössler, Ajdacic, & Klein, 2009; Gilmer et al., 2005; Murphy & Byrne, 2012), that is, they persist for at least 2 years. Since the DSM-5, this chronic course of depression has been classified as ‘persistent depressive disorder’ (PDD) (APA, 2013), which is distinguished from non-persistent forms of depression, which have a more episodic course. The latter includes the former diagnoses of a major depressive episode and recurrent major depressive disorder, hereafter referred to as episodic depression. Research has shown that people with persistent depressive disorder, hereafter referred to as persistent depression, are less likely to benefit from psychotherapy (Thase, Reynolds, Frank, & Simons, 1994) and pharmacotherapy (Howland, 1991) than people with episodic depression. In addition, people with persistent depression have a higher hospitalization rate and a lower level of functioning, which also causes higher costs for the healthcare system (Gilmer et al., 2005; Klein, Schwartz, Rose, & Leader, 2000; Luppa, Heinrich, Angermeyer, König, & Riedel-Heller, 2007). Given this worse prognosis for people with persistent depression, it is vital to understand the psychopathological factors that underlie the poorer response to available treatments.

Research has shown that there are indeed important psychopathological differences between persistent and episodic depression. For example, the two subtypes showed qualitatively different symptom trajectories over a period of 10 years (Klein & Kotov, 2016). Furthermore, people with persistent depression, as compared to people with episodic depression, more often have dysfunctional cognitions (Riso, Du Toit, & Blandino, 2003), have more difficulty to express their own emotions (van Randenborgh et al., 2012), and show a higher degree of emotional, cognitive, and behavioral avoidance (Brockmeyer, Kulessa, Hautzinger, Bents, & Backenstrass, 2015).

Based on these differences between the two subtypes of depression, we sought to further investigate factors that may have the potential to explain why patients with persistent depression respond less to treatments than patients with episodic depression. To this end, we focused on whether the two subtypes of depression differ in how they use novel positive information to alter established expectations. This interest draws from recent research that has demonstrated that depression in general is associated with a reduced update of negative beliefs by novel positive information (Everaert, Bronstein, Cannon, & Joormann, 2018; Korn, Sharot, Walter, Heekeren, & Dolan, 2014; Kube, Rief, Gollwitzer, Gärtner, & Glombiewski, 2019). Furthermore, it has been proposed that an important mechanism that may underlie the persistence of negative expectations is the cognitive devaluation of novel positive information, for example, through questioning the information’s validity, referred to as cognitive immunization (Kube, Schwarting, Rozenkrantz, Glombiewski, & Rief, 2020; Rief & Joormann, 2019). Preliminary empirical evidence supports this suggestion, as research has shown that the degree to which people with depression engage in a devaluation of novel positive information determines how much they alter their expectations in response to positive information (Kube et al., 2019; Kube, Rief, Gollwitzer, et al., 2019).

Following previous theoretical considerations (Barrett, Quigley, & Hamilton, 2016; Kube et al., 2020; Rief & Joormann, 2019), we hypothesize that the persistence of negative expectations and the engagement in cognitive immunization is more pronounced in people with persistent depression than in people with episodic depression. In doing so, we distinguish between expectations of psychotherapeutic treatment (e.g. ‘Psychotherapy will help me overcome my problems’) and expectations that refer to future life events (e.g. ‘Nothing positive will happen to me’). With regard to the former, we predict the treatment expectations of people with persistent depression, relative to those of people with episodic depression, to be more resistant to change because they more often experienced treatment failures in the past (Howland, 1991; Klein et al., 1999; Thase et al., 1994). Therefore, they may have more reason to doubt positive information on psychotherapy and engage in cognitive immunization, accordingly. With respect to expectations of future life, we expect the differences between the two subtypes of depression to be smaller, yet we predict the degree of expectation change still to be lower in people with persistent depression than in in people with episodic depression. We believe so because previous research has suggested that there is more pronounced avoidance behavior and social withdrawal in people with persistent depression (Brockmeyer et al., 2015; Jäger & Brakemeier, 2014; McCullough, 2003), and this lack of exploring might make their expectations more immune to change as they are less often challenged by disconfirming experiences.

In our view, focusing on (the lack of) expectation change and the cognitive processes that underlie it is promising to understand why people with persistent depression respond less to psychotherapy than people with episodic depression, because many psychotherapeutic interventions quite heavily rely on providing patients with novel positive experiences, such as behavioral experiments, behavioral activation, or emotion regulation (Dimidjian et al., 2006; Dobson & Hamilton, 2003; Rief & Glombiewski, 2016). If people with persistent depression had more difficulty than people with episodic depression in accepting and integrating novel positive experiences, this would explain why they benefit less from psychotherapeutic treatments, as experiential learning is a central component of most psychotherapeutic interventions (Craske, Treanor, Conway, Zbozinek, & Vervliet, 2014; Kube, Glombiewski, & Rief, 2019; Rief et al., 2022).

Accordingly, the present research aimed to test two hypotheses: first, that people with persistent depression adjust their expectations of psychotherapeutic treatment less than people with episodic depression in response to positive information on psychotherapy; and second, that people with persistent depression alter expectations of future life events less than people with episodic depression. These hypotheses were examined in an experimental study in which participants watched video recordings of other patients who reported on how they benefitted from psychotherapy. Moreover, we investigated whether the two subtypes of depression also differed in their engagement in cognitive immunization against positive information on psychotherapy, as well as other cognitive factors, namely, negative interpretations of positive information and deficient recall of it. These factors were chosen because negative interpretation bias (Everaert, Podina, & Koster, 2017) and deficient recall of positive information (Ellwart, Rinck, & Becker, 2003; Gotlib, Jonides, Buschkuehl, & Joormann, 2011) have been shown to be prevalent in depression, and we aimed to examine their importance in episodic versus persistent depression.

## Methods

This study is part of a larger research project, which sought to examine the role of cognitive immunization in expectation change in depression. The results of this manipulation irrespective of depression subtype are described elsewhere in detail (Kube, Rapo, Glombiewski, & Rief, 2025). In this article, we will focus on whether people with persistent depression responded differently to this manipulation than people with episodic depression. No further articles are planned using this data set. Participants from this study did not take part in any additional experiment.

### Ethics

The study was approved by the local ethics committee of the Department of Psychology at the university where the study was conducted (reference number LEK-190_2019). It was conducted in accordance with ethical standards as laid down in the 1964 Declaration of Helsinki and its later amendments. All participants gave written informed consent.

### Transparency and openness

The hypotheses, methods, and the analysis plan of the umbrella project were pre-registered (https://aspredicted.org/65B_3L2) on July 21, 2021, prior to data collection which began on August 11, 2021. In terms of openly sharing the data and the analysis code, we opted against fully sharing it through data repositories after extensive discussions for the following reason. We worried that highly potent data-analytic algorithms could be able to de-anonymize the patients’ identity with a certain probability when taking into account that it is publicly known where the study was conducted and, accordingly, in which geographical area the patients live, and which mental disorder was confirmed. Yet, the data and the analysis code will be made available to other researchers upon reasonable request.

### Participants

Participants were recruited between August 2021 and April 2024 from a German university outpatient clinic and several private psychotherapy practices in the surrounding. The key inclusion criterion was meeting the criteria of a major depressive disorder (ICD-10: F32.0-F32.2, F33.0-F33.2) or dysthymia (F34.1) as the primary diagnosis, as ascertained by a structured clinical interview. The Structured Clinical Interview for DSM-5 (SCID) was used in 94.2% of all cases to determine the diagnosis (Beesdo-Baum, Zaudig, & Wittchen, 2019). Notably, the SCID for DSM-5 allows to distinguish between persistent depressive disorder and episodic major depression. In 4.5% of the participants, the German ‘Diagnostisches Kurz-Interview bei psychischen Störungen’ (Mini-DIPS) was used as the diagnostic interview (Margraf, 1994). Since the Mini-DIPS does not distinguish between persistent and episodic depression according to DSM-5, it was followed by an additional interview that was specifically developed to assess whether people meet the criteria of a persistent depressive disorder (Klein, Backenstraß, & Schramm, 2018). In two patients (1.3%), another unspecified clinical interview was conducted, which too was followed by the interview for persistent depressive disorder. The diagnostic interviews were conducted by psychotherapists in training under the supervision of a licensed psychotherapist or by psychology master students, who were specifically trained in the SCID and supervised by a licensed psychotherapist. Further inclusion criteria were: patients had to be still in the diagnostic phase or at the very beginning of their psychotherapy (no more than 5 sessions); be at least 18 years old; and be fluent in German. Exclusion criteria were: psychotic symptoms and bipolar affective disorder. For their participation, participants received financial compensation (15 EUR). A total of 161 patients were included, of whom 156 could be included for the final analyses (see the supplementary material for a detailed description of the exclusions). This sample size was above the minimum sample size of *N* = 149, which was required according to the preregistered a-priori power analysis (expected effect size: *f* = 0.275; power = .80; *α* = .05; groups: 4; numerator df: 3).

According to the DSM-5 criteria, 65.4% met the criteria of an episodic major depression, and 34.6% met the criteria of a persistent depressive disorder. In terms of comorbidities, 37.8% had at least one comorbid mental disorder, and 12.2% had two comorbid diagnoses. The most frequent comorbid diagnoses were social anxiety disorder (10.3%), generalized anxiety disorder (7.7%), substance use disorder (5.1%), somatoform disorder (3.2%), panic disorder (2.6%), posttraumatic stress disorder (1.3%), obsessive-compulsive disorder (0.6%), hyperkinetic disorder (0.6%), and attention-deficit hyperactivity disorder (0.6%), and other anxiety disorders (4.5%). The mean BDI-II score in this sample was 28.78 (*SD* = 9.79), reflecting moderate to severe depressive symptoms (Beck, Steer, Ball, & Ranieri, 1996). About one third (32.7%) of the sample took antidepressant medication, of which Sertraline (7.7%) was the most frequently used antidepressant, followed by Escitalopram (5.1%) and Citalopram (3.2%). The other 67.3% did not take any psychopharmacological medication.

In terms of sociodemographic variables, participants’ age was between 18 to 70 years (*M* = 34.72, *SD* = 13.28), 67.3% reported to be female, 30.8% male, and three persons (1.9%) reported their gender to be diverse. As the highest educational degree, 39.2% had the school leaving examination (German ‘Abitur’ or ‘Fachabitur’), 30.8% had primary education, 21.8% had a university degree, 1.9% were still at school, and 6.4% had other educational degrees. In terms of employment status, 44.9% were employees, 22.4% were students, 6.4% were unemployed, 5.8% were in training, 2.6% were self-employed, 1.9% were still at school, 1.3% were civil servants, and 14.7% reported to have another status (e.g. being on parental leave, disabled, homemaker).

Of note, we deliberately did not examine a healthy control sample since previous research has repeatedly shown that depression is related to difficulties in using novel positive information to update negative beliefs (Deng et al., 2022; Everaert et al., 2018; Kube, Rief, Gollwitzer, et al., 2019). The engagement in cognitive immunization against novel positive information appears to specific to people with depression but does not occur in healthy people (Kube & Glombiewski, 2021, Kube & Glombiewski, 2022; Kube, Glombiewski, Gall, et al., 2019). In addition, the investigation of change in psychotherapy expectations might be perceived as odd by people who are mentally healthy and not seeking psychotherapeutic help.

### Procedure and stimuli

At the beginning, participants indicated their baseline expectations of psychotherapeutic treatment as well as their baseline expectations of future life events. Next, they were presented with videos from four patients, who were in fact amateur actors, as developed and validated by Rapo, Rief, and Kube (2023) (In the previous study by Rapo et al. (2023), we originally used videos of 5 characters including a man of about 30 years. In this pretest, however, participants fed back that they perceived the study as lasting very long and they recommended removing one character to shorten the duration of the study. We took this feedback seriously because the sample from this pretest was a sample of patients with major depression as well, and so our intention was to lower the burden related to participating in the study given the frequently reported problems concentrating in people with major depression. The video of the young man was removed based on the data from the pretest as it was perceived as being least suitable). Each of these four patients first reported on their symptoms and their current life situation at the beginning of their psychotherapy before reporting in another video from the end of their treatment on how psychotherapy helped them overcome these problems. The four patients shown in the videos varied with regard to a number of clinical variables to cover some of the most important manifestations of depression: character 1: anhedonia, along with problems concentrating, feelings of worthlessness, and suicidal ideation; character 2: depression with intense somatic symptoms such as headache, muscle tension, insomnia, and fatigue; character 3: seasonal recurrent depression with listlessness, social withdrawal, and changes in appetite; character 4: chronic depression with a pronounced loss of interest, psychomotor retardation, and escapism. Furthermore, the characters varied in terms of sociodemographic variables, such as age (25–61 years), gender (three female actresses versus one male actor), occupation (student versus employee versus pensioner versus civil service), family status (in a couple relationship versus single mum versus married versus divorced), and immigration history (one person with versus three persons without immigration history). All participants watched all videos and the order in which they were presented was randomized. Specifically, participants were presented with the first video of a randomly selected character before watching the second video of this character. Subsequently, they were randomly presented with the first video of another character, before watching its second video, and so forth. The videos from the beginning of treatment were between 2:30 and 5:01 minutes long, and the videos from the end of treatment had a length of between 2:07 and 4:10 minutes. In the supplementary material, we present the full list of details regarding the four characters and their reports (see Supplementary Table S1).

### Experimental conditions

In the cognitive immunization-inhibiting condition, participants were instructed to focus on similarities between themselves and the characters from the videos. This was supposed to make it difficult for participants to disregard the persons from the videos’ positive experiences with undergoing psychotherapy. To check whether participants actually followed that instruction, they were asked to note similarities they identified in an open text field. Participants from the cognitive immunization-promoting condition, on the other hand, were instructed to focus on and note down differences between themselves and the characters from the video. This was supposed to ease the engagement in cognitive immunization because paying attention to differences in the characters from the video may prompt the belief that their situations are hardly comparable, such that participants may think that they may not benefit from psychotherapy as much as the persons from the videos did.

In the distraction control group, participants were instructed to focus on the physical appearance of the person in the video and noted these aspects in open text fields, accordingly. This condition was used as an active control group to be able to test whether really the content of the cognitive immunization manipulation – and not just any unspecific instruction which interrupts the procedure – makes a difference to expectation change. Finally, the no-instruction control group did not receive any instruction before watching either of the videos. Further details of the experimental manipulations and their reasoning are reported elsewhere (Kube et al., 2025).

### Measures

#### Expectations for psychotherapeutic treatment

Participants’ expectations of psychotherapeutic treatment were assessed using the ‘Milwaukee Psychotherapy Expectation Questionnaire’ (MPEQ) by Norberg, Wetterneck, Sass, and Kanter (2011). The MPEQ comprises 13 items, which relate to two factors: role expectations (e.g. ‘I expect my therapist will provide support’) and outcome expectations (e.g. ‘After therapy, I will be a much more optimistic person’). The items are rated on an 11-point scale from 0 (= ‘not at all’) to 10 (= ‘very much so’) and we report the arithmetic mean of the MPEQ, which can range between 0 and 10, accordingly. In the present study, Cronbach’s alpha of the MPEQ was *α* = .83 at baseline and *α* = .86 after watching the videos.

#### Expectations of future life events

We used the Future Event Questionnaire (FEQ) by Miranda and Mennin (2007) to assess participants’ expectations of experiencing positive and negative life events. In the instruction of the FEQ, participants received the following information: ‘For each of the following events, please rate how likely you think it is that you will experience it at some point in the future’. Next, participants were presented with 17 positive (e.g. ‘Having a successful career’) and 17 negative future events (e.g. ‘Get the blame for something going wrong’), which cover a range of important life domains, such as social relationships, occupation, and finance. Participants’ task was to indicate the likelihood of each event to happen to them on a five-point Likert scale ranging from 1 (‘not at all likely’) to 5 (‘very likely’). To compute the FEQ mean score (ranging from 1 to 5), participants’ ratings for the negative events are reversely scored, such that a high FEQ score reflects positive future expectations. Cronbach’s *α* of the FEQ was .90 at baseline and .93 after watching the videos.

#### Cognitive immunization

We used the previously developed ‘Cognitive Immunisation Against Other People’s Experiences’ (CIA-OPE) scale (Rapo et al., 2023) to assess whether participants engage in cognitive immunization against the other patients’ reports. The 5-item scale used in the present study comprises three items that assess whether participants question the comparability of their own situation and that of the characters from the videos (e.g. ‘The problems the person mentioned in the video apply to me as well’). Another two items assess whether participants question the reports’ credibility and authenticity (e.g. ‘The report of this person is authentic’). The items of the CIA-OPE were assessed after the second video of each of the four characters and were rated on a 7-point Likert-scale from 1 (‘totally disagree’) to 7 (‘totally agree’). For all four characters, Cronbach’s alpha was *α* = .83. For the sub-items referring to the comparability of participants’ own situation and the person from the video, Cronbach’s alpha was α_comparability_ = .85 and for the items referring to the credibility of the reports, it was α_credibility_ = .90.

#### Interpretations

We also assessed whether participants had the propensity to interpret the video reports negatively, using a 7-item scale developed previously (Rapo et al., 2023). With this scale, we assessed whether participants interpreted the patients’ reports as an indication of how psychotherapy improved their mood and their life situation – or whether they were inclined to interpret the patients’ reports more negatively (e.g. ‘The person made the impression that they still felt depressed after the therapy’). The items were rated on a 7-point Likert scale from 1 (‘totally disagree’) to 7 (‘totally agree’). Cronbach’s alpha across all four characters was *α* = .91.

#### Recall

In addition, we assessed whether participants correctly recalled the contents from the videos. To this end, they were presented with five statements for each of the four characters (resulting in a total of 20 statements), some of which were correct, meaning that they corresponded to something the person from the video stated, whereas other statements were incorrect. The total score for all four characters can range from 0 to 20, with a value of 20 reflecting that there was not any error in recall.

#### Depressive symptoms

Depressive symptoms were assessed using the second edition of the Beck’s Depression Inventory (BDI-II), which includes 21 items assessing depressive symptoms on a 4-point scale ranging from 0 to 3 (Beck et al., 1996). The sum score ranges between 0 and 63, and lower values indicate fewer depressive symptoms.

### Statistical analyses

Because participants could only continue with the survey if they entered all values, there were no missing values for the experimental sessions. Data screening was conducted according to the recommendations of Tabachnick and Fidell (2019). For the main analysis, we performed a time (2: before versus after watching the other patients’ reports) by diagnosis (persistent versus episodic depression) repeated-measures analysis of variance (ANOVA) using participants’ treatment expectations (MPEQ) as the dependent variable. A similar ANOVA was performed to examine whether the two subtypes of depression differ in changing their expectations of future life events. Type-I error levels were set at 5%. All analyses were performed using SPSS version 29.

## Results

### Differences between persistent and episodic depression in sociodemographic, clinical, and baseline study variables

Participants with persistent depression did not significantly differ from participants with episodic depression in terms of age, *t*(96.458) = −1.370, *p* = .174, *d* = −0.240, 95% CI [−0.571, .091], gender distribution, χ^2^(2) = 5.052, *p* = .080, educational degrees, χ^2^(7) = 7.346, *p* = .394, employment status, χ^2^(7) = 3.318, *p* = .854, and German as the native language, χ^2^(1) = 2.958, *p* = .085.

In terms of clinical variables, the two subtypes of depression did not differ in their BDI-II scores, *t*(154) = −0.356, *p* = .722, *d* = −0.060, 95% CI [−0.390, .270] and the presence of comorbid secondary diagnoses, χ^2^(11) = 11.943, *p* = .368. However, people with persistent depression significantly differed from people with episodic depression in the distribution of tertiary comorbid diagnoses, χ^2^(8) = 19.538, *p* = .012: participants with persistent depression more frequently met the criteria of generalized anxiety disorder as the tertiary diagnosis (5 versus 0 cases), whereas participants with episodic depression more frequently met the criteria of other anxiety disorders as the tertiary diagnosis (0 versus 5 cases). The percentage of participants who met the criteria of any tertiary diagnosis was not significantly different between episodic (16.7%) and persistent depression (9.8%), though, χ^2^(1) = 1.555, *p* = .212 (see Table 1). The two subtypes of depression did not significantly differ in the intake of antidepressant medication either, χ^2^(1) = 0.708, *p* = .400.

**Table 1.** Differences between persistent and episodic depression in sociodemographic, clinical, and baseline study variables

Variable	Persistent depressive disorder (*n* = 54)	Episodic major depression (*n* = 102)	Significant differences
Age, M (SD)	36.80 (14.38)	33.62 (12.60)	*p* = .174, *d* = −0.240
Gender, N (%)			χ^2^(2) = 5.052, *p* = .080
Male	22 (40.7%)	26 (25.4%)	
Female	32 (59.3%)	73 (71.6%)	
Diverse	0	3 (2.9%)	
Education level, N (%)			χ^2^(7) = 7.346, *p* = .394
Primary education	16 (29.6%)	35 (34.3%)	
School leaving examination	23 (42.6%)	38 (37.2%)	
University degree	10 (18.5%)	24 (23.5%)	
Other	5 (9.3%)	5 (4.9%)	
Employment status, N (%)			χ^2^(7) = 3.318, *p* = .854
At school	0	3 (2.9%)	
In training	3 (5.6%)	6 (5.8%)	
University student	12 (22.2%)	23 (22.5%)	
Employed	26 (48.1%)	44 (43.1%)	
Self-employed	1 (1.9%)	3 (2.9%)	
Civil servant	0	2 (2.0%)	
Unemployed	3 (5.6%)	7 (6.9%)	
Other	9 (16.7%)	14 (13.7%)	
BDI-II sum score, M (SD)	29.17 (11.46)	28.58 (8.83)	*p* = .722, *d* = −0.060
Comorbid secondary diagnosis, N (%)			χ^2^(1) = 0.299, *p* = .584
any	22 (40.7%)	37 (36.3%)	
none	32 (59.3%)	65 (63.7%)	
Comorbid tertiary diagnosis, N (%)			χ^2^(1) = 1.555, *p* = .212
Any	9 (16.7%)	10 (9.8%)	
None	45 (83.3%)	92 (90.2%)	
Antidepressant medication, N (%)			χ^2^(1) = 0.708, *p* = .400
Yes	20 (37.0%)	31 (30.3%)	
No	34 (63.0%)	71 (69.7%)	
FEQ T1, M (SD)	2.86 (0.50)	2.94 (0.51)	*p* = .347, *d* = 0.159
MPEQ T1, M (SD)	7.94 (1.09)	8.33 (0.95)	*p* = .022, *d* = 0.390

*Note:* N, number; M, mean; SD, standard deviation; BDI-II, Beck’s Depression Inventory Revised; FEQ, Future Event Questionnaire; MPEQ, Milwaukee Psychotherapy Expectation Questionnaire; T1, baseline assessment. Note that here we present the data for the presence of any (versus none) secondary or tertiary diagnosis, whereas the main text presents differences in the frequency distribution for all specific diagnoses, which is why the statistical information differs between main text and this table.

In terms of baseline study variables, participants with persistent depression did not differ from participants with episodic depression in baseline expectations of future life events, *t*(154) = 0.943, *p* = .347, *d* = 0.159, 95% CI [−0.172, 0.489]. However, people with persistent depression had significantly less positive treatment expectations than people with episodic depression, *t*(154) = 2.318, *p* = .022, *d* = 0.390, 95% CI [0.057, 0.719], reflecting a small to medium effect. Table 1 presents all sociodemographic, clinical, and baseline study data of patient with persistent versus episodic depression.

### Differences between persistent and episodic depression in change of treatment expectations

**Figure 1.**
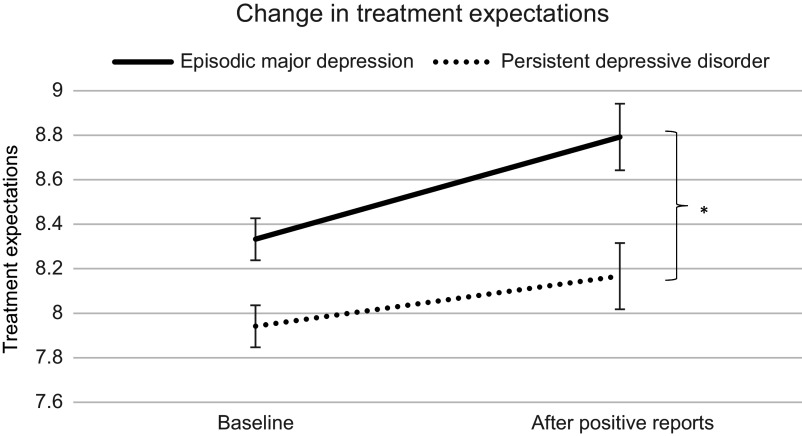
Results of the differences between persistent and episodic depression in changing expectations of psychotherapeutic treatment. The results show that participants with episodic depression adjusted their treatment expectations in response to other patients’ reports of the positive effects of psychotherapy more than participants with persistent depression. * *p* < .05, error bars reflect the standard error of the mean.

There was a significant time by diagnosis interaction, *F*(1, 154) = 5.716, *p* = .018, η_p_^2^ = .036, 90% CI [.003, .095], indicating that people with persistent depression (*M* = 0.22, *SD* = 0.57) adjusted their expectations less than people with episodic depression (*M* = 0.46, *SD* = 0.59), as illustrated in Figure 1. This reflects a small to medium effect, *d* = 0.402, 95% CI [0.069, 0.735]. This effect also remained significant when controlling for the severity of depressive symptoms (BDI-II sum scores) as a covariate, *F*(1, 153) = 5.625, *p* = .019, η_p_^2^ = .035, 90% CI [.003, .095]. When distinguishing between role expectations and outcome expectations (i.e. the two factors of the MPEQ), we found that the time by diagnosis interaction was significant particularly for change in outcome expectations, *F*(1, 154) = 6.935, *p* = .009, η_p_^2^ = .043, 90% CI [.006, .106], whereas it was not significant for change in role expectations, *F*(1, 154) = 1.553, *p* = .215, η_p_^2^ = .010, 90% CI [0, .051]. With respect to the latter, however, there was a significant main effect of diagnosis, *F*(1, 154) = 15.192, *p* < .001, η_p_^2^ = .090, 90% CI [.030, .166], indicating that people with persistent depression had significantly lower role expectations than people with episodic depression (reflecting a medium effect, *d* = 0.603).

**Figure 2.**
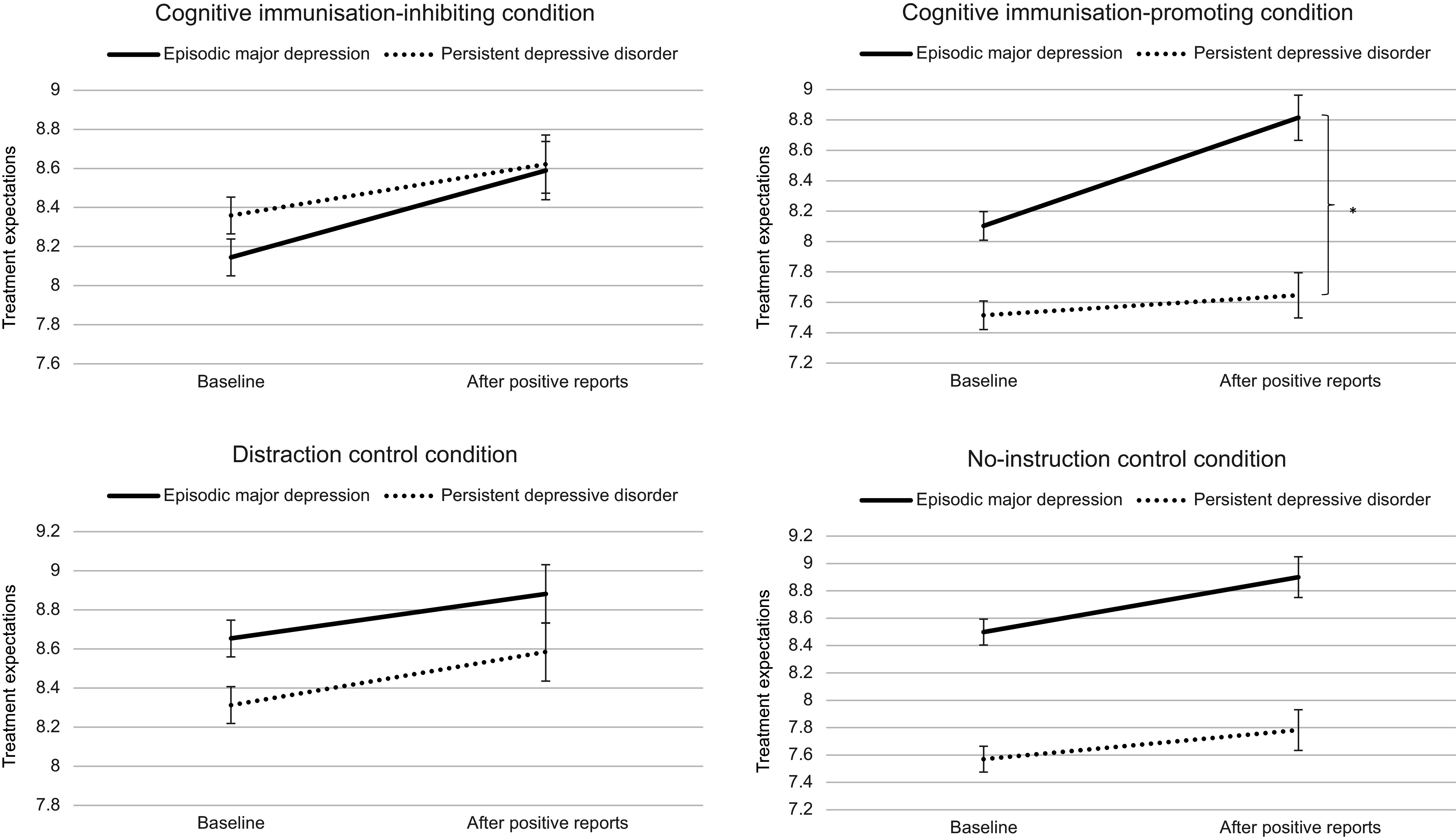
Results of the differences between persistent and episodic depression in change of expectations for psychotherapeutic treatment, separately for the four experimental conditions. As compared to participants with episodic depression, change in treatment expectations in participants with persistent depression was particularly low in the cognitive immunization-promoting condition. * *p* < .05, error bars reflect the standard error of the mean.

When adding the experimental condition (focusing on differences versus similarities of participants acting patients) as another between-subjects factor, the time by diagnosis interaction remained significant (*p* = .022). In addition, there was a significant diagnosis by condition interaction, *F*(3, 148) = 3.010, *p* = .032, η_p_^2^ = .057, 90% CI [.003, .113] (Again, this effect remained significant when controlling for the severity of depressive symptoms, *F*(3, 147) = 3.000, *p* = .033, η_p_^2^ = .058, 90% CI [.003, .113]). Post-hoc tests showed that only in the cognitive immunization-promoting condition, participants with persistent depression showed significantly less expectation change than participants with episodic depression, *t*(37) = 2.271, *p* = .029, *d* = 0.833, 95% CI [0.084, 1.571], reflecting a large effect, whereas the two subtypes of depression did not differ in expectation change in the other experimental conditions (all *p*-values > .290), as depicted in Figure 2. The time by diagnosis by condition interaction was not significant, *F*(3, 148) = 1.643, *p* = .182, η_p_^2^ = .032, 90% CI [0, .075].

### Differences between persistent and episodic depression in expectations of future life events

**Figure 3.**
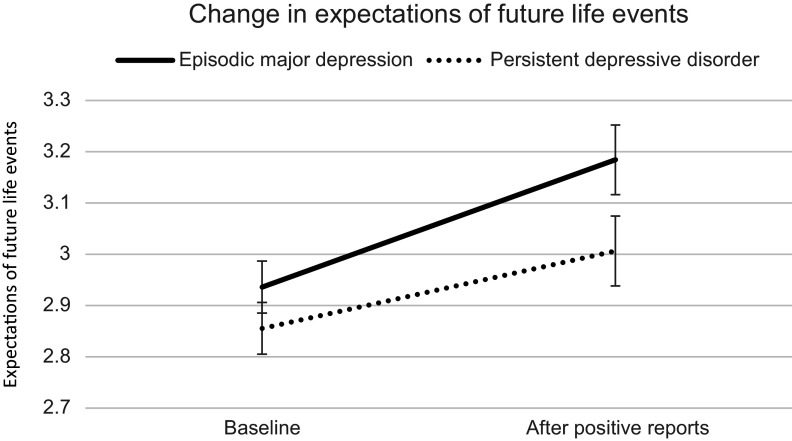
Differences between persistent and episodic depression in changing expectations of future life events. The results show that participants with episodic depression adjusted their expectations descriptively somewhat more than participants with persistent depressive disorder, but this effect was not statistically significant. Error bars reflect the standard error of the mean.

The ANOVA indicated that the time by diagnosis interaction was not significant, *F*(1, 154) = 2.165, *p* = .143, η_p_^2^ = .014, 90% CI [0, .059]. The main effect of diagnosis was not significant either, *F*(1, 154) = 2.214, *p* = .139, η_p_^2^ = .014, 90% CI [0, .059]. However, there was a significant main effect of time*, F*(1, 154) = 36.352, *p* < .001, η_p_^2^ = .191, 90% CI [.105, .278], indicating that overall, participants’ treatment expectations were more positive after watching the other patients’ reports (*M* = 3.12, *SD* = 0.60) than before (*M* = 2.91, *SD* = 0.51), see Figure 3.

When adding the experimental condition as another between-subjects factor, the time by diagnosis by condition interaction was not significant, *F*(3, 148) = 1.285, *p* = .282, η_p_^2^ = .025, 90% CI [0, .064].

### Differences between persistent and episodic depression in cognitive factors

People with persistent and episodic depression did not significantly differ in cognitive immunization, *t*(154) = 0.334, *p* = .739, *d* = 0.056, 95% CI [−0.274, 0.386], neither with respect to questioning the comparability, *t*(154) = 0.920, *p* = .359, *d* = 0.155, 95% CI [−0.176, 0.485], nor with respect to questioning the authenticity of the characters’ situations, *t*(154) = −1.010, *p* = .314, *d* = −0.170, 95% CI [−0.500, 0.161]. They did not significantly differ in their endorsement of negative interpretations, *t*(154) = −0.969, *p* = .334, *d* = −0.163, 95% CI [−0.493, 0.168], and deficient recall either, *t*(154) = −0.888, *p* = .376, *d* = −0.149, 95% CI [−0.479, .181].

## Discussion

In view of evidence suggesting that people with persistent depression respond less to available treatments than people with episodic depression (Keller & Boland, 1998; Kocsis, 2003), the present research sought to investigate differences between the two subtypes of depression in terms of their psychopathology. Specifically, we examined whether people with persistent depression differ from people with episodic depression in the extent to which they use novel positive information to adjust treatment expectations and expectations of future life events in response to positive information on psychotherapy. The results show that patients with persistent depression had not only overall lower treatment expectations than patients with episodic depression, but their treatment expectations were also more resistant to change. This was particularly pertinent in expectations regarding the outcomes of psychotherapy, indicating that participants with persistent depression showed less change in their expectations of improvement through psychotherapy than participants with episodic depression. By contrast, the two subtypes did not differ in changing role expectations, although here again people with persistent depression had overall lower expectations. The results also show that the lacking update of treatment expectations in people with persistent depression was particularly pronounced in the cognitive immunization-promoting condition. Contrary to treatment expectations, the two subtypes of depression did not differ in their update of expectations of future life events.

The finding that treatment expectations are more resistant to change in patients with persistent depression than in patients with episodic depression is interesting and relevant, in view of research showing that low treatment expectations are a risk factor of not benefitting from psychotherapy (Constantino, Vîslă, Coyne, & Boswell, 2018; Greenberg, Constantino, & Bruce, 2006). In terms of underlying mechanisms, the present study focused on the role of cognitive immunization. Consistent with theoretical predictions (Kube et al., 2020; Rief & Joormann, 2019), we found that changes in treatment expectations were almost completely blocked in people with persistent depression if they underwent the cognitive immunization-promoting condition. This suggests that for people with persistent depression, integrating positive information about psychotherapy can be hindered if they become aware of differences between themselves and others. As a result, they may question the usefulness of such positive information and change their expectations less than they otherwise would. However, since this was not reflected by differences in the cognitive immunization scale, it is not entirely clear whether the results regarding changes in treatment expectations were really driven by this factor. It may be that the scale to assess cognitive immunization was just not good enough to capture this process. Another possible explanation, though, would be that the differences between the two subtypes of depression in changing treatment expectations were caused by another, unmeasured process, such as more pronounced negative affect (Kube, Kirchner, Gärtner, & Glombiewski, 2023) or attention bias (Everaert, Duyck, & Koster, 2014; Keller, Leikauf, Holt-Gosselin, Staveland, & Williams, 2019). However, interpretation bias and deficient recall can be ruled out as underlying mechanisms with a fair degree of certainty because the two subtypes of depression did not differ on these measures.

Another perspective on the finding that treatment expectations were more resistant to change in people with persistent depression is to consider patients’ treatment history. According to this view, it is understandable that people with persistent depression are more hesitant to adjust their treatment expectations, given their frequent treatment failures and their often long history of suffering (Klein et al., 1999; McCullough, 2003). Thus, we suggest to interpret these results such that subjective disappointments with previous treatments made people with persistent depression more sceptical about the promised positive effects of psychotherapy. This interpretation is in line with recent qualitative findings indicating that people with depression often aim to avoid future disappointments, at the expense of holding on to negative beliefs (Kube & Rauch, 2025). It also accords well with the rationale of a psychotherapeutic treatment that was specifically developed for persistent depression, the cognitive behavioral analysis system of psychotherapy (CBASP), where the therapeutic relationship is specifically used to alter patients’ treatment expectations (Brakemeier et al., 2015; Keller et al., 2000; Schramm et al., 2017). However, it is important to note that we did not collect more specific data on patients’ previous treatments, so this interpretation must remain speculative at this point until further testing. It should also be noted that in absolute terms, people with persistent depression were not pessimistic regarding the effects of psychotherapy. Rather, the descriptive values of their treatment expectations suggest that their expectations were just less positive than those of people with episodic depression.

An additional perspective that may be worth exploring is that of developmental psychopathology. Specifically, research has shown that people with persistent depression often have a history of childhood maltreatment (Brakemeier et al., 2018; Brown et al., 2013), which is a risk factor for important aspects of the psychopathology of persistent depression, such as impaired social cognition and interpersonal problems (Struck, Gärtner, Kircher, & Brakemeier, 2021). It is also discussed as a predictor of worse course of depression (Nanni, Uher, & Danese, 2012; Nelson, Klumparendt, Doebler, & Ehring, 2017) and treatment outcomes (Bausch et al., 2020; Harkness, Bagby, & Kennedy, 2012; Nanni et al., 2012). In view of the prevalence of childhood maltreatment in people’s histories and its detrimental psychological consequences, it is understandable that many of the people who are diagnosed with persistent depressive disorder have an early onset of the disorder (Agosti, 1999; Brockmeyer et al., 2015; Lizardi et al., 1995). Thus, aside from differences in treatment history, the etiology of persistent depression appears to be quite different from episodic depression, which is more often related to an unsuccessful adaptation to life events (Andrews & Thomson, 2009; Durisko, Mulsant, & Andrews, 2015; Nesse, 2000). Accordingly, although the aforementioned factors were not assessed in the present study, this developmental perspective may explain why the expectations of people with persistent depression are more resistant to change: They have been formed over a longer period of time, resulting in greater confidence in them. As a result, people with persistent depression may be less open to integrating other people’s reports into their expectations (Barrett et al., 2016; Kube et al., 2020; Rief & Joormann, 2019). The disregard of such reports may be particularly pronounced if the differences between the other person and oneself are salient, which may explain why people with persistent depression had particular difficulty revising their expectations in the cognitive immunization-promoting condition.

Aside from treatment expectations, it is not so surprising that people with persistent depression did not differ from people with episodic depression in changing expectations of future life events, because this type of expectation has not been considered a core feature of persistent depression that distinguishes it from episodic depression. Rather, since persistent depression has been theorized to be linked to negative interpersonal expectations (e.g. ‘Others will treat me badly’), it is conceivable that the two subtypes of depression would differ in the degree of change in interpersonal expectations (Kirchner et al., 2025; McCullough, 2003).

### Clinical implications

The finding that the treatment expectations of people with persistent depression, as compared to episodic depression, are not only generally lower but also more resistant to change is important from a clinical point of view, as treatment expectations have been considered an important predictor of psychotherapy outcomes (Constantino et al., 2018; Greenberg et al., 2006). Thus, the risk of treatment failure might be higher in persistent depression than in episodic depression because people with persistent depression are less likely to expect to benefit from psychotherapy. On the other hand, given the treatment failures of many people with persistent depression in the past, it may actually be realistic for them to be more skeptical about the effects of psychotherapy. Nevertheless, in order to enhance the likelihood of benefitting from psychotherapy, it may be important to take additional efforts to increase patients’ treatment expectations. To this end, it may be important for therapists to make sure that they are perceived as warm and competent when informing about the effects of psychotherapy, since the therapist’s warmth and competence have been shown to modulate the degree to which people update their psychotherapy outcome expectations (Seewald & Rief, 2023, 2024). Furthermore, it may be worthwhile to use the therapeutic relationship to establish positive treatment expectations over the course of the first sessions of psychotherapy, as done in CBASP (McCullough, 2003). However, not only for psychotherapists but also for practitioners who refer patients to psychotherapy, it may be important to improve patients’ treatment expectations from the outset. In addition, the current results must not be interpreted in the sense that only patients with persistent depression need their clinicians to engage with their treatment expectations. Rather, also patients with episodic depression, like presumably all other patients, need their therapists to address their treatment expectations. In addition, with regard to the results regarding cognitive immunization, it may be important to prevent patients with persistent depression from focusing on differences between themselves and others when informing them about the effects of psychotherapy. To this end, it may be helpful to have patients specify why such information on the effects of psychotherapy may also apply to them.

### Strengths and limitations

The present research was the first to systematically investigate differences between people with persistent and episodic depression in changing expectations in response to novel positive information, therein distinguishing between treatment expectations and expectations of future life events. To this end, we were able to use a relatively large data set from an outpatient sample. Further strengths can be seen in the careful diagnostic approach to distinguish between persistent and episodic depression, the assessment of a number of additional potentially relevant variables (i.e. clinical aspects and cognitive factors), and the preregistration of the umbrella project.

A significant limitation is that the question of the mechanisms underlying the differences between persistent and episodic depression in terms of changes in treatment expectations cannot be completely answered by the current data. Specifically, there is some indication that these differences may be related to the engagement in cognitive immunization, but the data does not fully support this suggestion. In addition, we did not collect more specific data on patients’ treatment histories, which is why our interpretation of the results with regard to possible negative previous treatment experiences in people with persistent depression must remain speculative. A further limitation is that we focused only on expectations of future life events as an empirically supported example of depression-specific expectations (Miranda & Mennin, 2007), but we acknowledge that other disorder-specific expectations are important as well, such as expectations of social rejection (Kirchner et al., 2023; Kirchner, Schummer, Krug, Kube, & Rief, 2022) and expectations regarding mood regulation (Backenstrass et al., 2006; Catanzaro, 1996).

With regard to the sample, we explained in the methods section why we opted against examining also a healthy control sample. However, we note that the absence of a healthy control sample may still be seen as a limitation, as the inclusion of a healthy control sample would have allowed us to examine whether people with persistent versus episodic depression respond differently to the manipulation of cognitive immunization as compared to healthy participants. Our reasoning was that this question cannot be well addressed with the current design focusing on treatment expectations, because the idea of seeking psychotherapeutic help would necessarily be hypothetical for healthy volunteers. However, we acknowledge that the inclusion of a healthy control sample would have been helpful to determine the extent to which depression in general contributed to the current results. Yet, this issue does not negate the differences between episodic and persistent depression, which was the main focus of the present article. Furthermore, in terms of sample size, we note that the study was sufficiently powered to examine differences between the two subtypes of depression in expectation change, but the power to uncover interactions with the experimental conditions may not have been sufficient, although there was indeed a significant time by diagnosis by condition interaction for treatment expectations. Moreover, we note as a further limitation that the degree of diversity of the videos presented was limited, although this was inevitable with only four characters. In addition, the fact that three of the four characters were female was not ideal in terms of gender balance.

## Conclusions

The present research demonstrates that in people with persistent depression, expectations of psychotherapeutic treatment are overall lower and more resistant to change than in people with episodic depression. This difference between the two subtypes of depression was particularly pronounced for psychotherapy outcome expectations, whereas it was smaller for role expectations. In addition, the results suggest that the resistance to change of treatment expectations in persistent depression is particularly strong when patients become aware of differences between themselves and other patients who report to have benefitted from psychotherapy, as indicated by the effects of the cognitive immunization manipulation. Beyond treatment expectations, we found that people with persistent depression and people with episodic depression did not differ in adjusting expectations of future life events. Further research is needed to identify strategies through which more positive treatment expectations in people with persistent depression can be established.

## Supporting information

Kube et al. supplementary material Kube et al. supplementary material

## Supplementary material

The supplementary material for this article can be found at http://doi.org/10.1017/S0033291725101530.
