# Supplementary material for: Differences between persistent and episodic depression in processing novel positive information
Source: Psychol Med. 2025 Sep 4;55:e261. doi: 10.1017/S0033291725101530 (PMC13040592; doi:10.1017/S0033291725101530)
Supplement: Kube et al. supplementary material [file S0033291725101530sup001.docx]

**Supplementary Material**

**Exclusion of participants**

One person had to be excluded because there turned out to be a diagnostic failure, that is, the person did not have MDD as the primary diagnosis. Similarly, one person had to be excluded because it turned out that they had completed already seven psychotherapy sessions. Another two participants had to be excluded due to technical problems during the study completion, such that the videos could not be played. In addition, one person had to be excluded as a statistical outlier (> 3 *SD*s above the mean on the dependent variable). Thus, we had a final sample size of *N* = 156 patients with major depression as the primary diagnosis.

Table S1

*List of all characters used in the video-taped patient reports*

| **Character** | **Sociodemographic characteristics** | **Clinical problems before therapy** | **Pictures** |
| --- | --- | --- | --- |
| Anna-Lena | - Female - 25 years old - Studies media and communication - In a couple relationship | - Anhedonia - Social withdrawal - Problems concentrating - Feeling worthless | 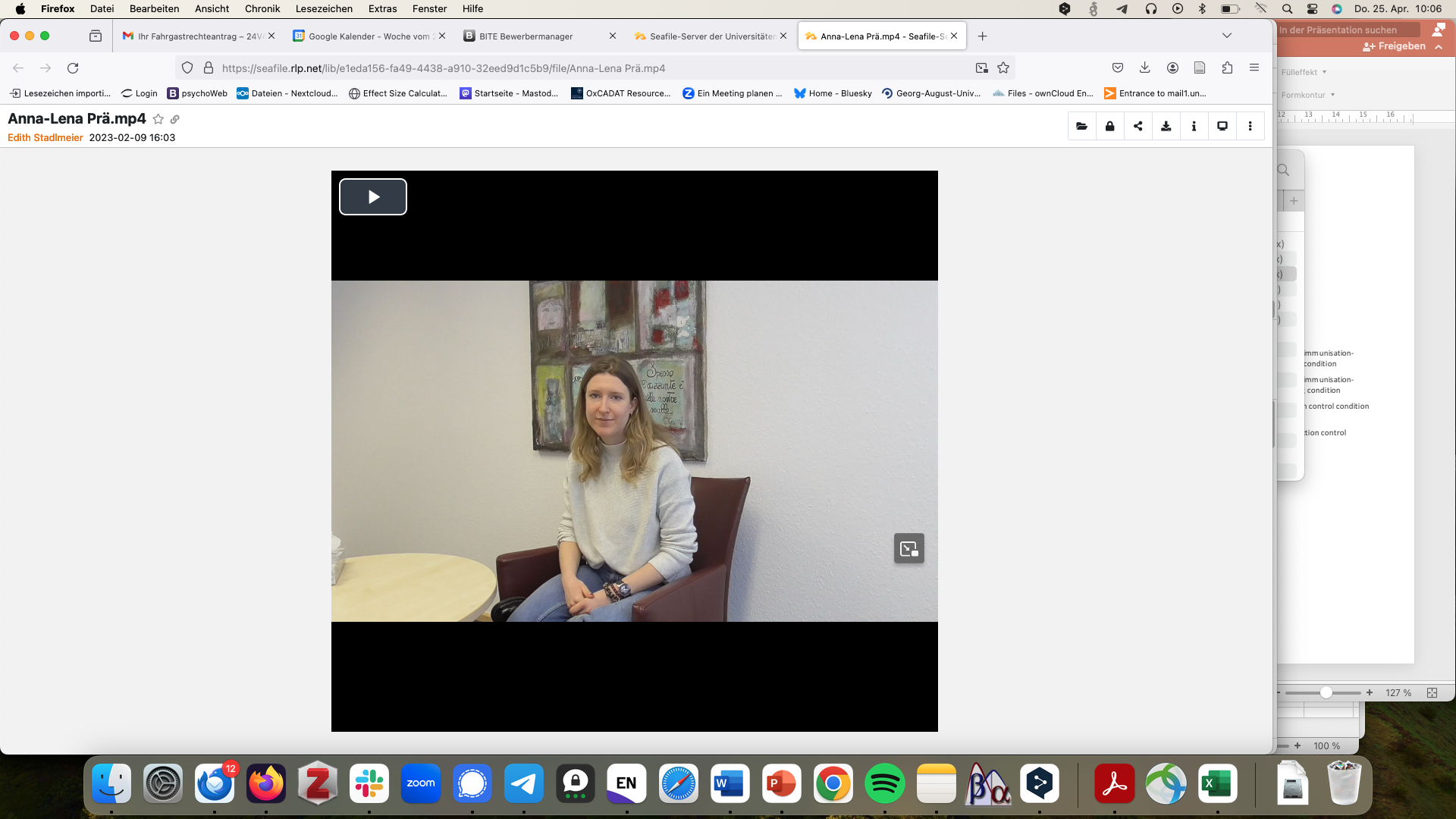 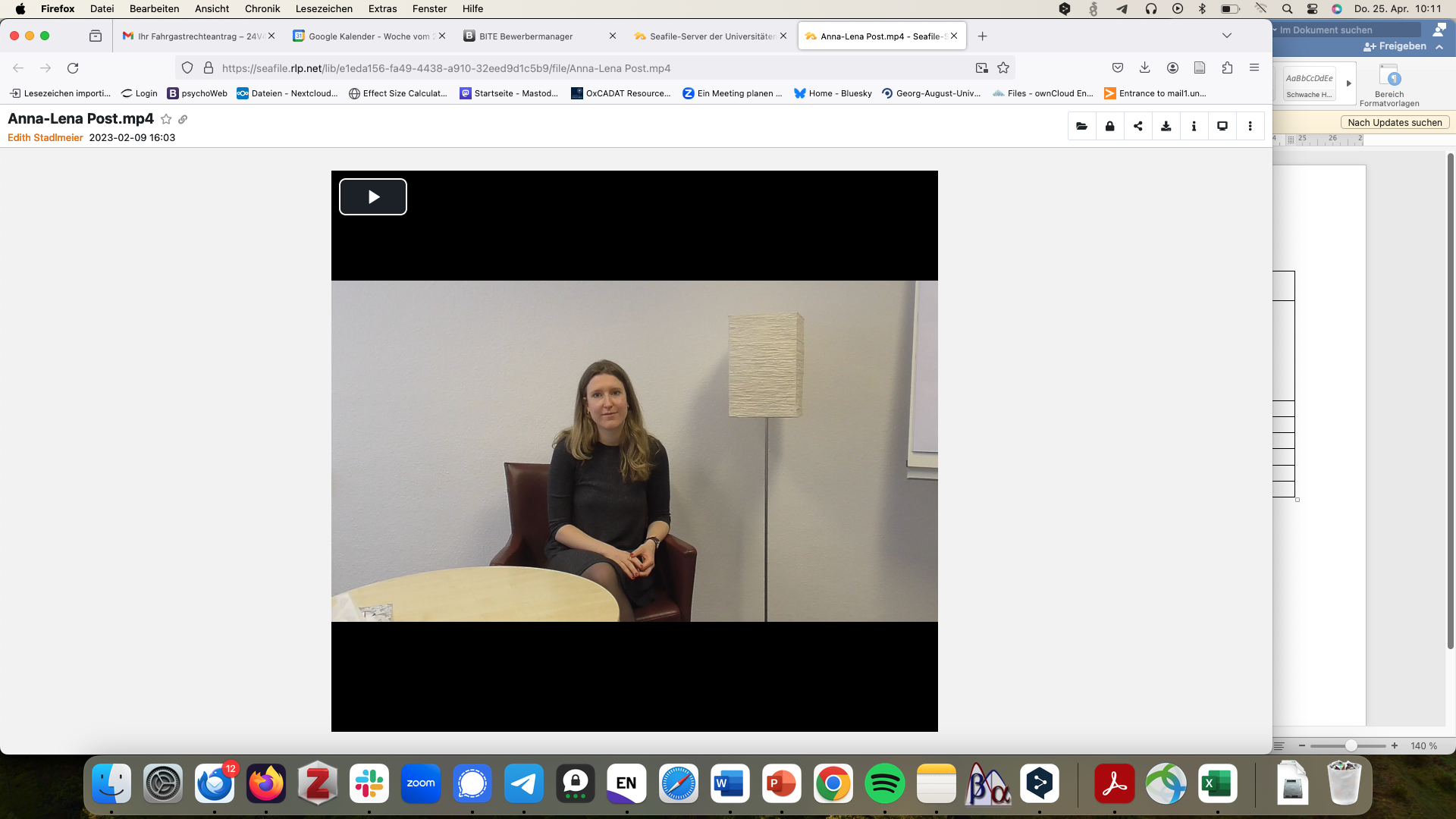  *Before therapy At the end of therapy* |
| Maria | - Female - 37 years old - Immigration history (Spain) - Single mom of two children - Most recent partner died one year ago - Financial problems despite full-time working | - Stressed out by managing her daily life - Intense somatic symptoms such as headache - Struggles with the diagnosis depression - Strong loss of interest, even in her children, although she feels highly responsible for them | 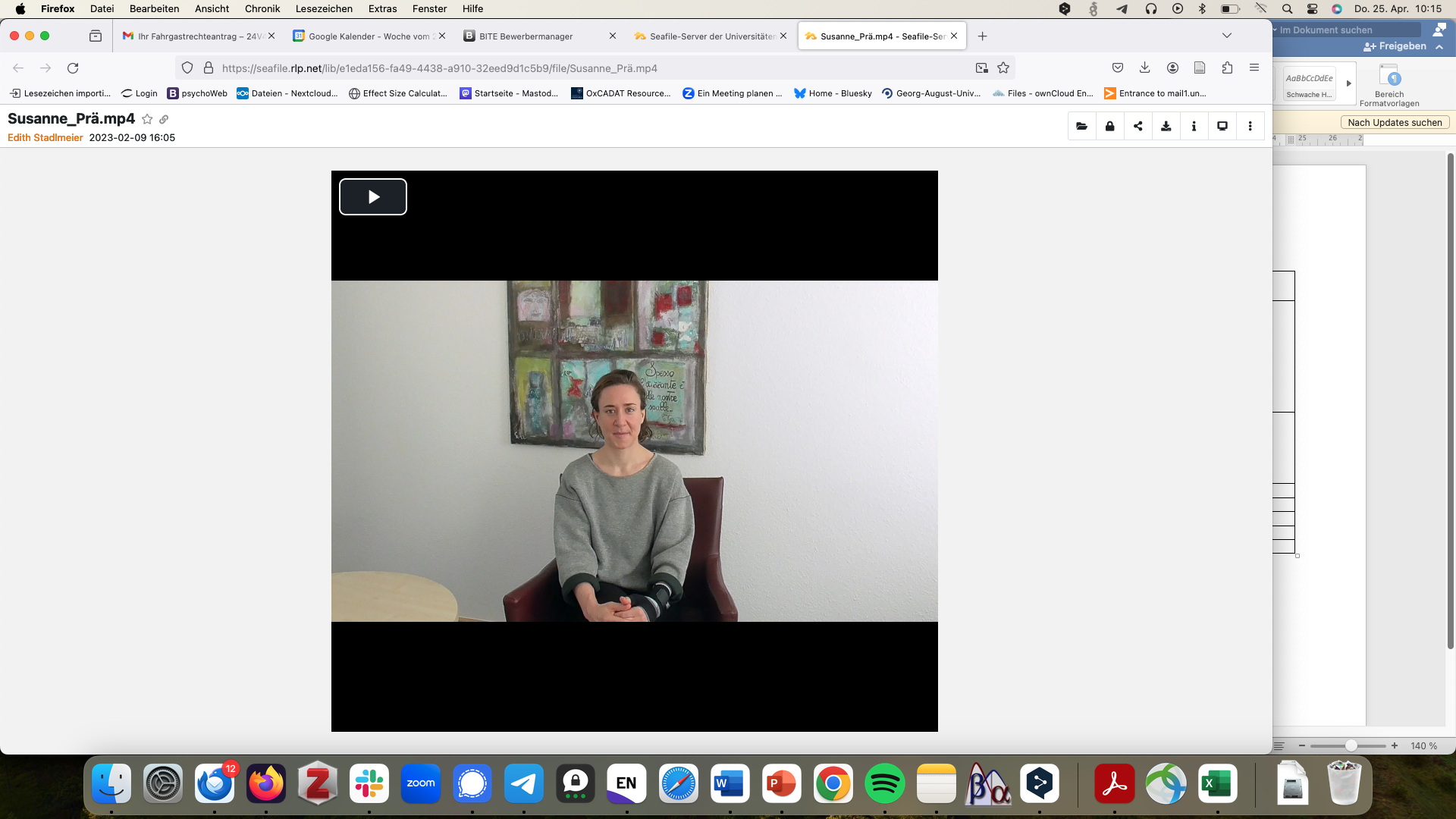 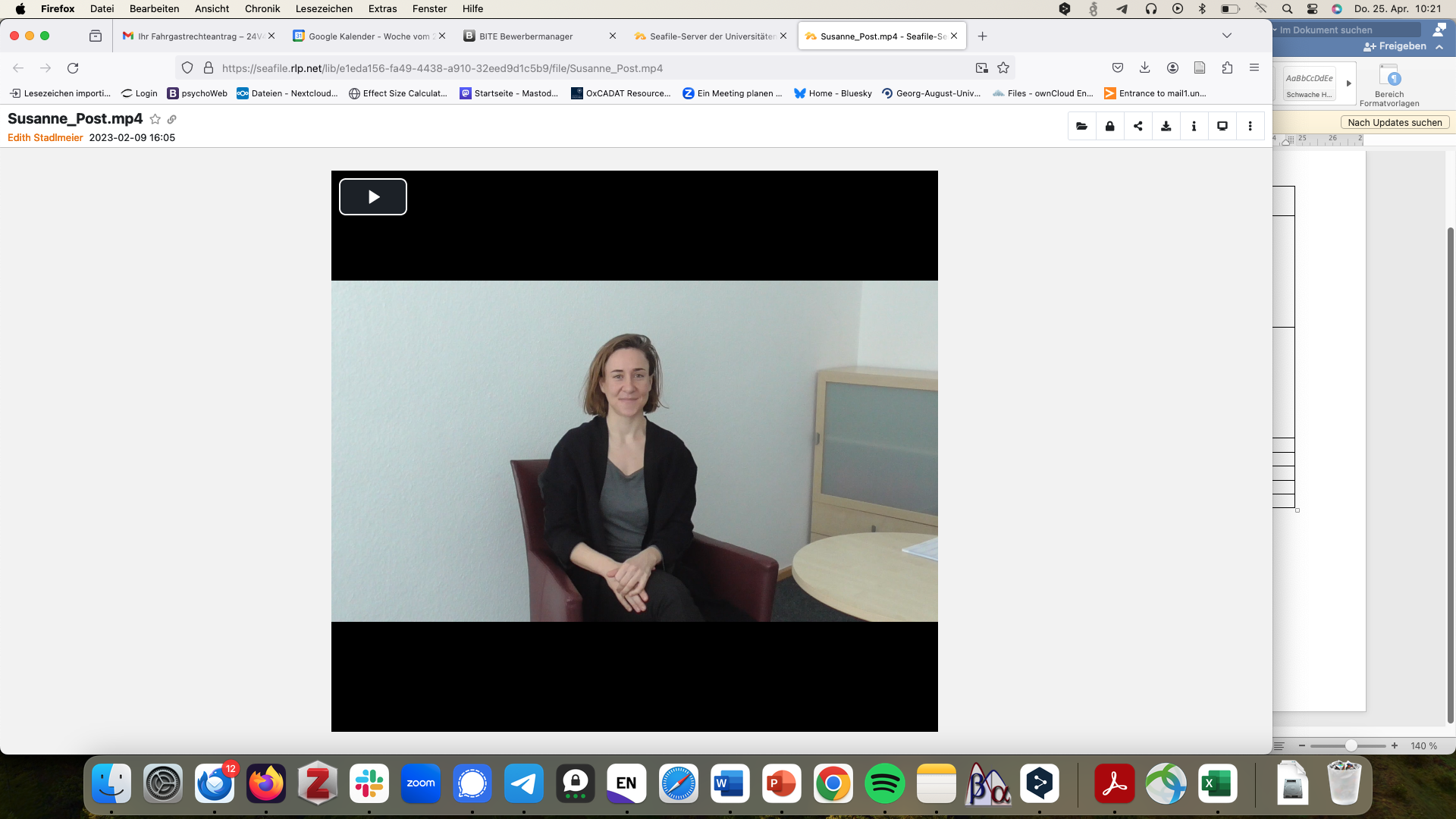  *Before therapy At the end of therapy* |
| Gabi | - Female - 55 years old - Married - Two adult children - Part-time working, husband pensioner | - Seasonal recurrent depression - Increased appetite - Loss of interest - Social withdrawal | 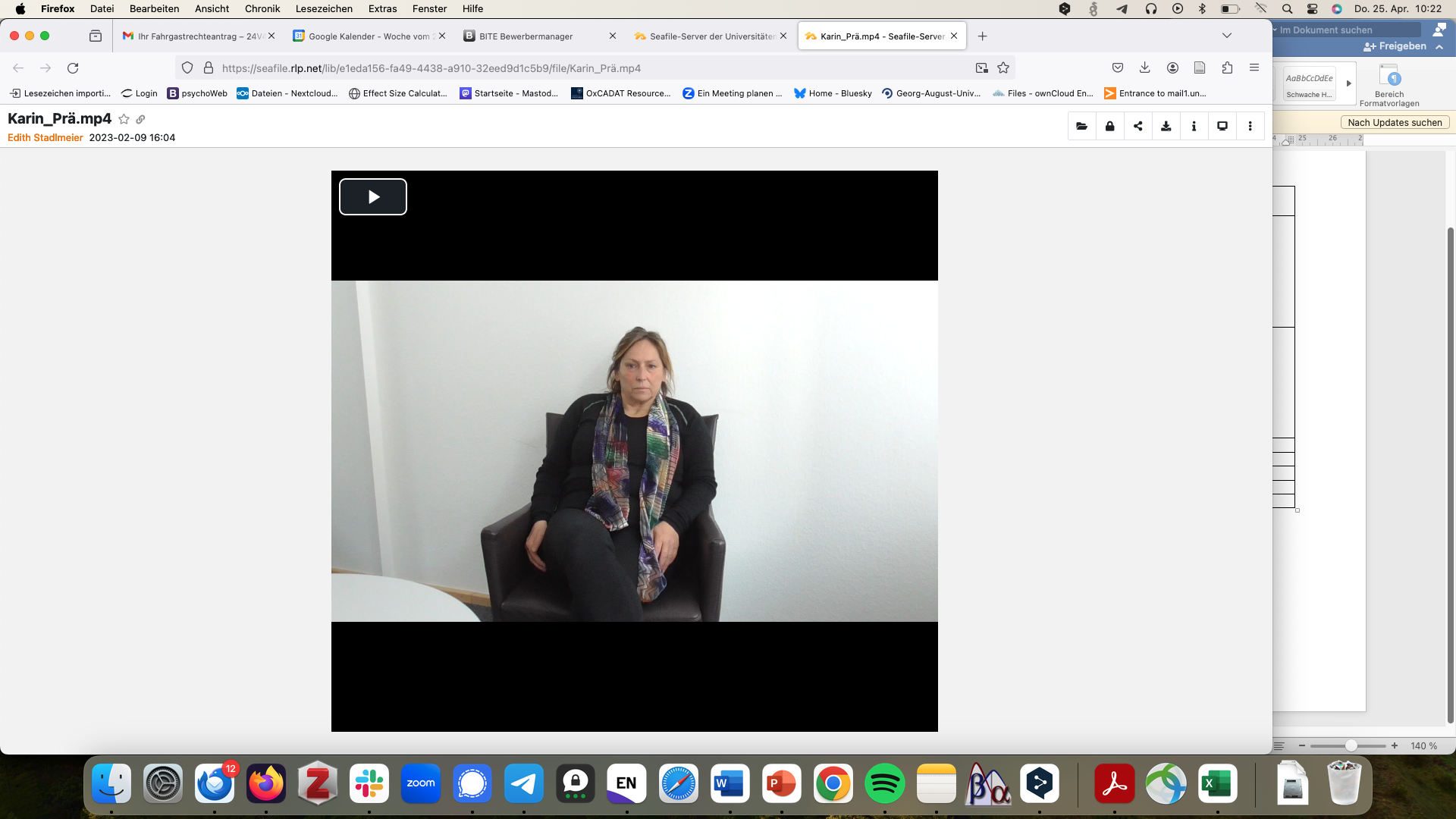 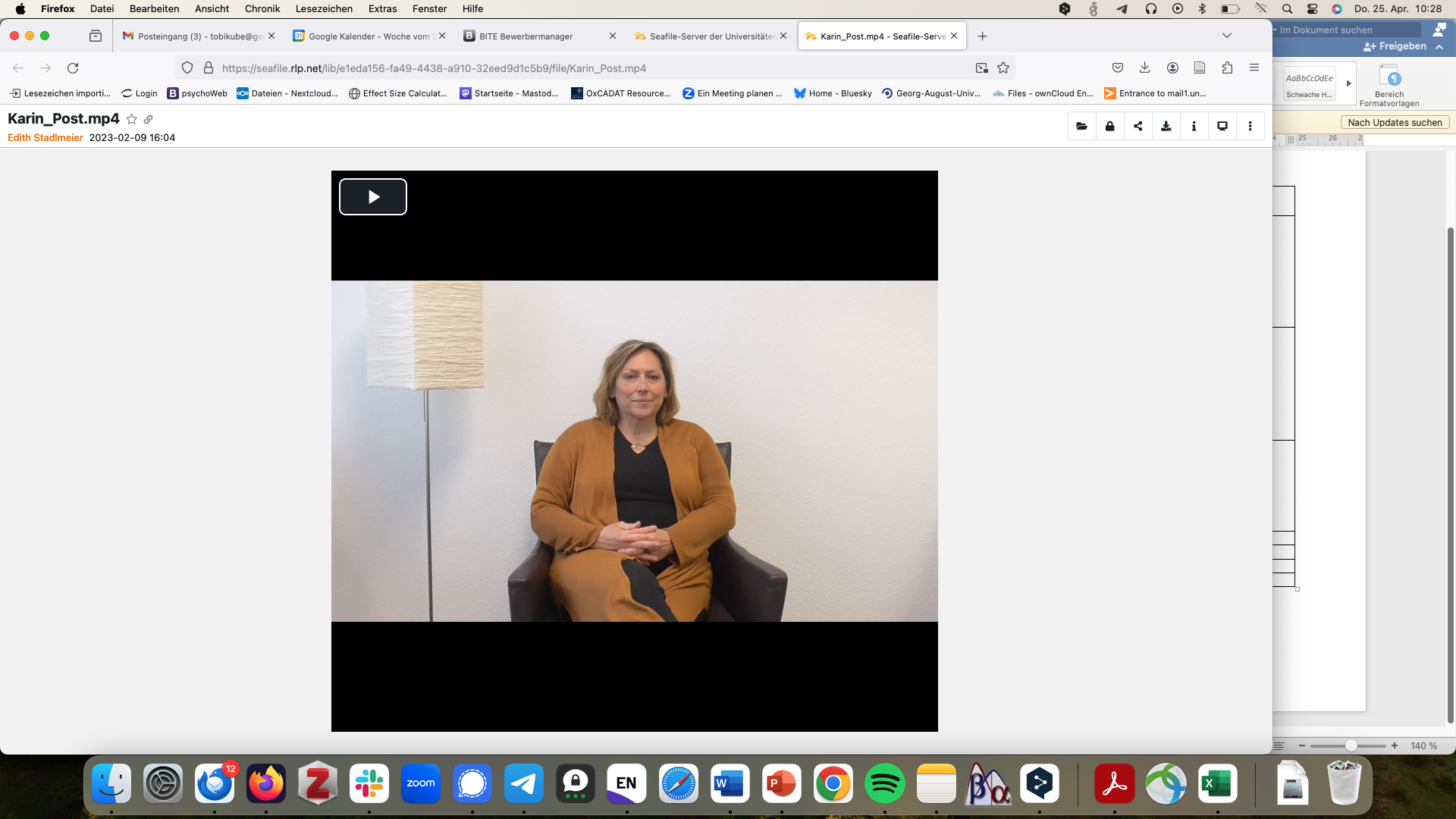  *Before therapy At the end of therapy* |
| Erwin | - Male - 63 years old - Recently divorced, no children - Works in civil service | - Chronic depression - Experiences his life as boring and pointless - Psychomotor retardation - Escapism | 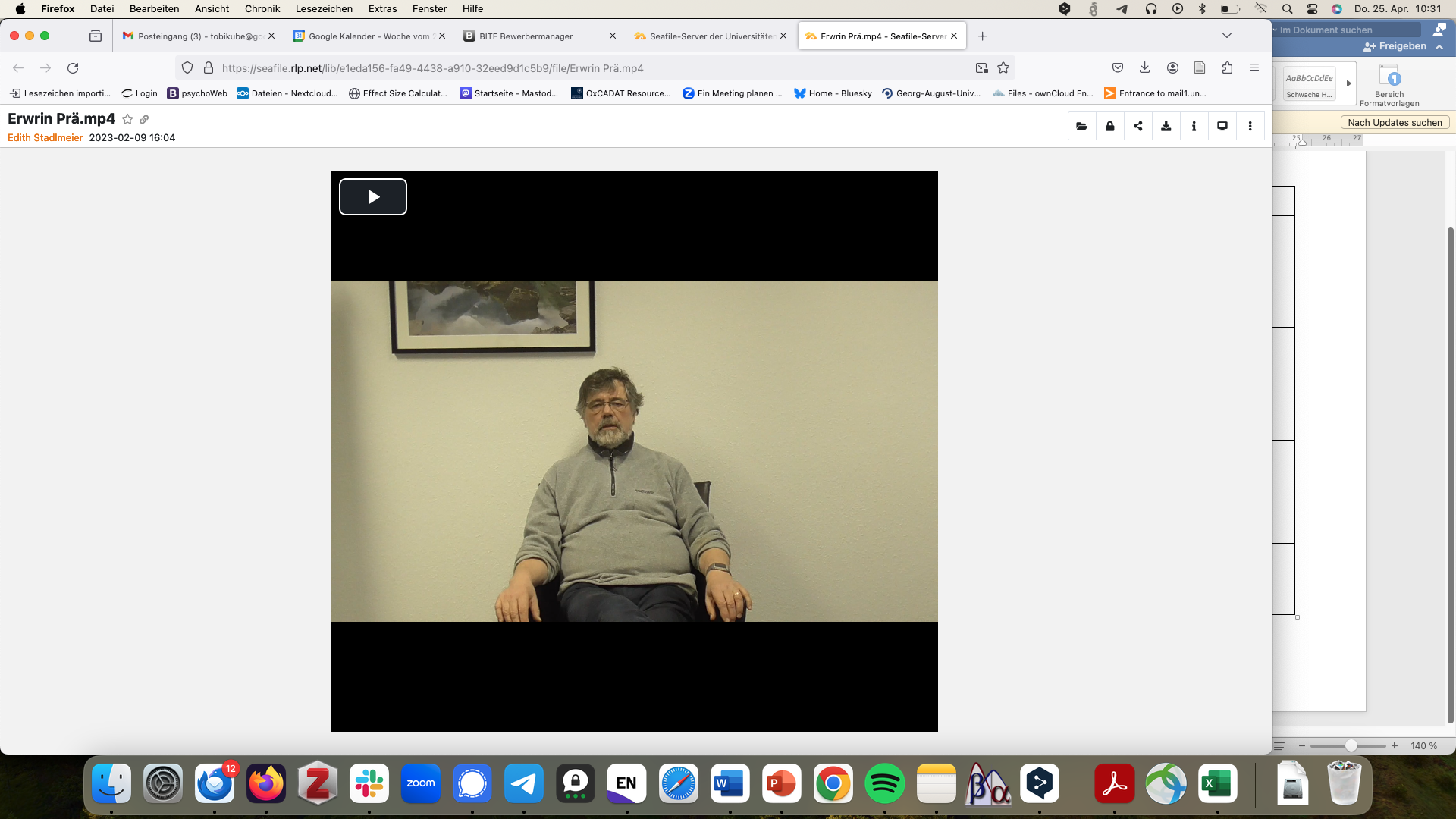 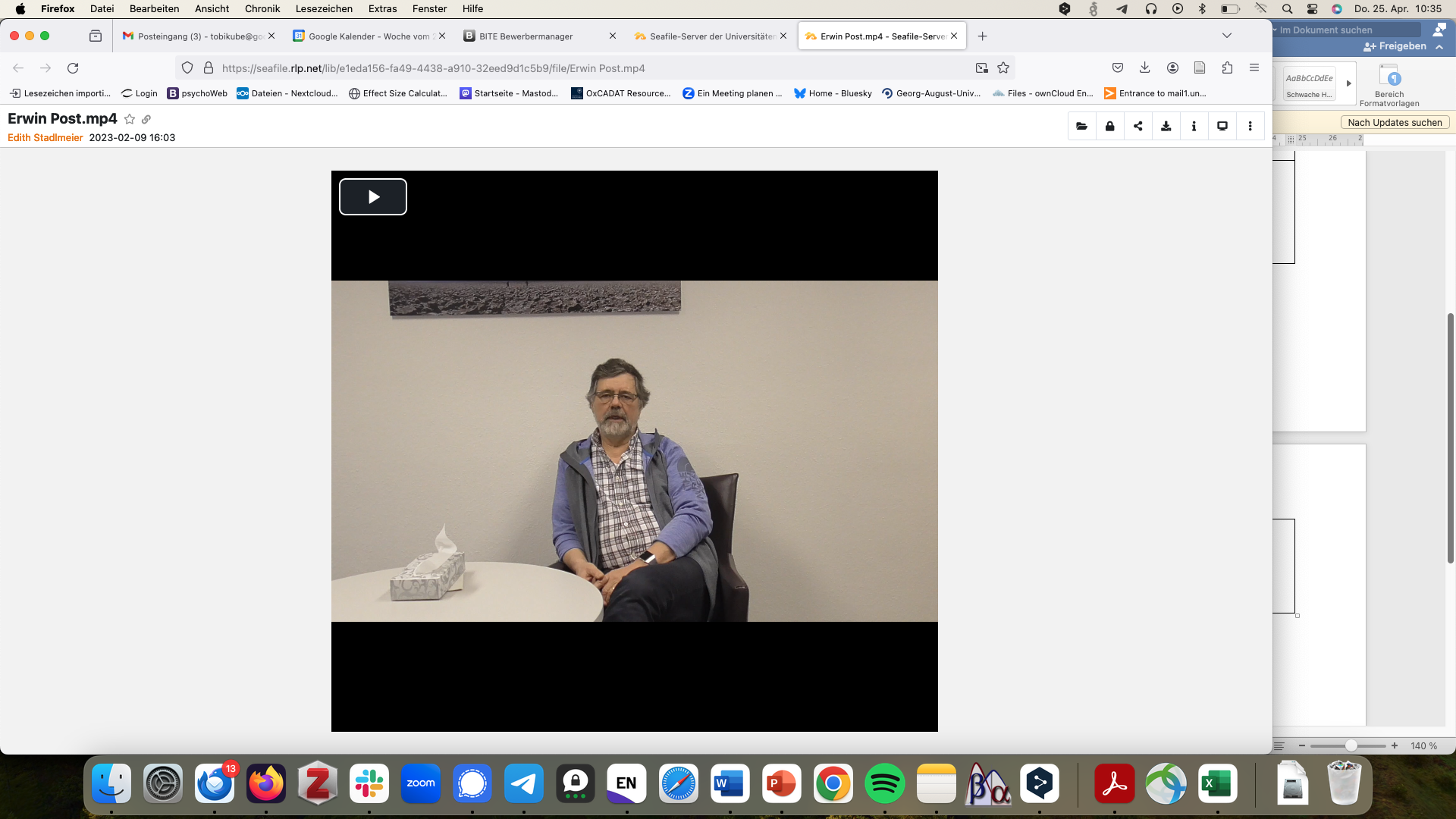  *Before therapy At the end of therapy* |
